# Lysosomal homeostasis at the crossroads of neurodegeneration

**DOI:** 10.1172/JCI199845

**Published:** 2026-04-01

**Authors:** Stefano De Tito, Sharon A. Tooze

**Affiliations:** 1 Navira Bio at; 2 The Francis Crick Institute, London, United Kingdom.

## Abstract

Lysosomes function as metabolic control centers that integrate degradation, nutrient sensing, and stress signaling. In neurons, which must maintain proteostasis and energetic balance throughout life, lysosomal homeostasis determines cellular resilience. Emerging evidence identifies lysosomal injury and defective repair as common denominators across neurodegenerative diseases. Damage to the lysosomal membrane caused by oxidative stress, lipid imbalance, or genetic mutations triggers a hierarchical quality control cascade. Early lesions recruit the endosomal sorting complex required for transport (ESCRT) machinery for mechanical resealing, while larger ruptures activate lipid-centered recovery modules. When repair fails, lysophagy eliminates irreparable organelles and a TFEB-dependent transcriptional program regenerates the lysosomal pool. These tightly coupled responses safeguard neurons from catastrophic proteostatic collapse. Their impairment, through mutations in lysosomal proteins, or through aging, produces the lysosomal fragility that underlies Alzheimer disease, Parkinson disease, amyotrophic lateral sclerosis/frontotemporal dementia, and Huntington disease. Crosstalk between lysosomes, mitochondria, and ER integrates local damage with systemic metabolic adaptation, while dysregulated lysosomal exocytosis and inflammation propagate pathology. Understanding how ESCRT complexes, lipid transport, and transcriptional renewal cooperate to preserve lysosomal integrity reveals unifying principles of neurodegeneration and defines molecular targets for intervention. Restoring lysosomal repair and renewal offers a rational path toward preventing neuronal loss.

## Introduction

The lysosome, an acidic membrane-bound organelle rich in hydrolytic enzymes, is now recognized as a dynamic signaling hub central to metabolism, quality control, stress adaptation, and inter-organelle communication (1–6). The membrane of the lysosome anchors key signaling complexes, including mammalian target of rapamycin complex 1 (mTORC1), AMP-activated protein kinase (AMPK), and transcription factor EB (TFEB), which link nutrient and energy status to global cellular programs (7, 8).

In neurons, lysosomal homeostasis is uniquely critical due to their postmitotic nature and extreme polarization (9, 10). Maintaining lysosomal acidity, enzyme content, and trafficking fidelity across long axons is an ongoing challenge. Neuronal lysosomes not only degrade cargo but also mediate synaptic remodeling and plasma membrane repair (11–13). Even subtle defects in lysosomal function can initiate progressive proteostatic and metabolic failure that culminates in neurodegeneration.

Genetic and pathological evidence firmly places lysosomal dysfunction at the center of neuronal loss (14–17). Recent lysosome immunopurification (Lyso-IP) studies revealed disease-associated accumulation of aggregation-prone proteins such as α-synuclein, tau, and TDP-43 that precedes overt membrane damage (18, 19). Mutations in lysosomal proteins that disrupt hydrolase trafficking, affect lysosomal membrane repair, or alter lipid metabolism are implicated in diverse neurodegenerative diseases (Table 1). In sporadic conditions, neurodegeneration, oxidative stress, lipid peroxidation, and chronic mTORC1 activation progressively weaken lysosomal integrity (10, 20–22).

When the lysosomal membrane becomes damaged and leaky, a process termed lysosomal membrane permeabilization (LMP), cells activate a hierarchical lysosomal quality control system (23, 24). Depending on the extent of damage, this system coordinates mechanical repair, lipid remodeling, selective clearance, and transcriptional regeneration (24–26). Successful recovery restores homeostasis, whereas failure results in inflammasome activation, mitochondrial dysfunction, and neuronal death (6, 10, 27, 28).

## Mechanisms of lysosomal damage and repair

**Figure 1:**
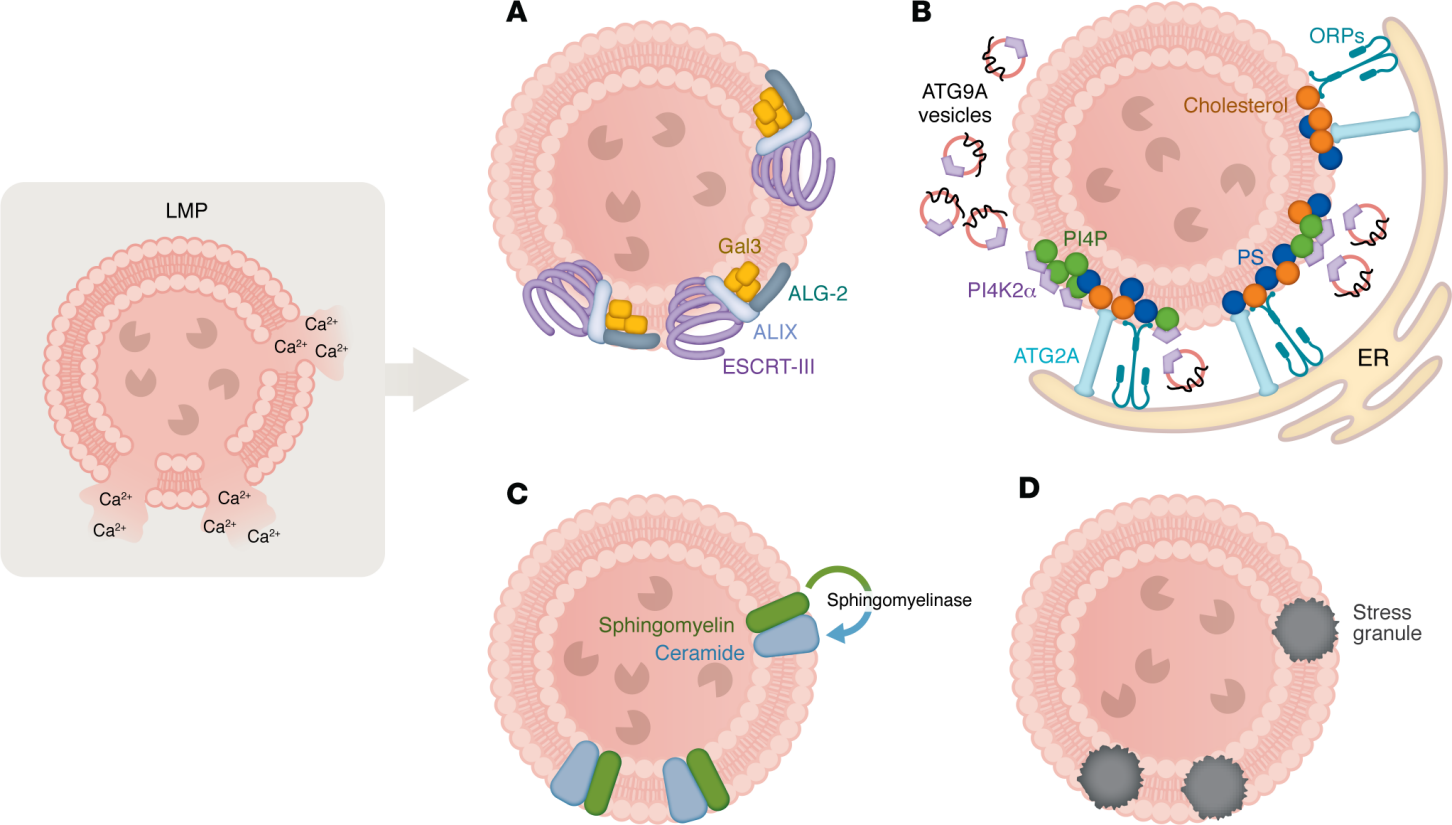
Mechanisms of lysosomal membrane repair. (**A**) The ESCRT machinery, recruited by galectin-3 (Gal3) and ALIX, polymerizes at rupture sites to reseal small pores. (**B**) The PI4K2A/ORP/ATG9A lipid-transfer axis forms ER-lysosome bridges that restore membrane lipid composition and curvature. ATG2 cooperates with ATG9A vesicles to deliver lipids for bilayer expansion. (**C**) Neutral sphingomyelinase–dependent conversion of sphingomyelin to ceramide promotes local fusion and sealing. (**D**) Under severe stress, protein-RNA condensates assemble into transient stress granule patches that shield lesions until structural repair completes. Together, these coordinated modules preserve lysosomal integrity and prevent cathepsin release.

LMP occurs along a continuum from transient nanoscopic pores to catastrophic rupture (29–31). Even limited permeabilization exposes luminal glycans to the cytosol, rapidly recruiting galectins-3, -8, and -9 (32–35). Galectins are a conserved family of cytosolic lectins containing carbohydrate recognition domains that bind β-galactoside–containing glycoconjugates. As most lysosomal membrane proteins are glycosylated, membrane disruption exposes their β-galactosides to the cytosol, where they are rapidly detected by galectins that serve as sentinels of lysosomal integrity (34). Galectin-3 interacts with the endosomal sorting complex required for transport (ESCRT) adaptor ALIX and TSG101 to nucleate ESCRT-III filaments that constrict and seal the membrane leak (33, 36). Local calcium efflux through the lesion activates ALG-2, which bridges ALIX to phospholipids and stabilizes ESCRT polymerization (37–39) (Figure 1). The AAA ATPase VPS4 subsequently disassembles ESCRT-III, recycling the subunits for additional rounds of repair (40, 41). In neurons, this process unfolds within seconds, preserving lysosomal integrity and preventing the leakage of cathepsins that would otherwise activate inflammasomes and induce mitochondrial depolarization (42, 43).

Concurrently, galectin sensors transduce metabolic signals that transiently suppress growth programs and promote autophagy. Galectin-8 assembles the GALTOR complex, which binds the Ragulator/Rag axis and inhibits mTORC1, thereby facilitating TFEB activation (34, 35). Galectin-9, through USP9X-dependent TAK1 ubiquitination, stimulates AMPK and broad autophagic responses (44, 45). Together, these events couple structural repair with metabolic reprogramming, ensuring that the cell prioritizes restoration over growth during lysosomal stress.

When membrane injury exceeds ESCRT capacity, a lipid-centered recovery module is engaged (46–50). Phosphatidylinositol 4-kinase type 2-α (PI4K2A) generates phosphatidylinositol-4-phosphate (PI4P) at the damaged surface, stimulating the phosphoinositide-initiated tethering and lipid-transport (PITT) pathway (48, 49) (Figure 1). PI4P recruits oxysterol-binding protein (OSBP) and the OSBP-related proteins (ORPs) ORP9, ORP10, and ORP11, which tether the lysosome to VAPA and VAPB on the ER, establishing a lipid-exchange bridge. Through these contacts, PI4P is transferred to the ER, while phosphatidylserine (PS) and cholesterol flow back to the lysosome. Furthermore, the lipid transfer protein ATG2 facilitates phospholipid flow from the ER to damaged lysosomes, promoting membrane expansion and restoration (49, 51). This bidirectional transfer restores bilayer asymmetry, curvature, and mechanical resilience. A pivotal element of this process is ATG9A, a transmembrane protein essential for autophagy (46, 52–55). ATG9A mainly resides in the Golgi and endosomes and cycles between other organelles, including the plasma membrane and lysosome (46, 50, 56). It has been shown to have lipid scramblase activity that is required for its function in autophagy (53, 55) and membrane repair (46, 57). ATG9A traffics from the *trans*-Golgi network to the damaged lysosome in response to injury (46, 50). ATG9A vesicles deliver PI4K2A to the lesion site, where newly synthesized PI4P creates microdomains to seal lysosomal membrane damage via lipid transfer of ORPs through ER-lysosome contact sites (46) (Figure 1). ARFIP2, a Bin/amphiphysin/Rvs homology (BAR) domain–containing protein that detects membrane curvature (58, 59), senses these PI4P-enriched zones (60), coordinating the lipid-transfer proteins to complete the resealing process. This ATG9A/PI4K2A/ARFIP2 axis has been defined as an autonomous repair route parallel to ESCRT. However, whether ESCRT- and lipid-mediated repair pathways operate sequentially, competitively, or in a damage-specific hierarchy remains an open question. Disruption of ATG9A trafficking or ARFIP2 sensing leads to alteration in lysosomal homeostasis, a phenotype similar to that observed altering vesicle trafficking in dopaminergic neurons of Parkinson disease (PD) models (61–63).

Sphingolipid metabolism further contributes to membrane recovery. Activation of sphingomyelin scramblases exposes sphingomyelin to the cytosolic leaflet, where neutral sphingomyelinase generates ceramide (64). Ceramide’s conical geometry promotes local fusion and sealing, while cholesterol imported from the ER through OSBP stiffens the membrane and reduces permeability (64–66). The interplay among PI4P, PS, ceramide, and cholesterol defines a lipid code that determines whether a damaged lysosome is repaired or removed (26) (Figure 1).

In parallel, recent evidence suggests that stress granules transiently associate with damaged lysosomes and may supply membrane components that limit leakage (67) (Figure 1). This stress granule–mediated sealing provides an auxiliary repair route that complements ESCRT- and lipid-driven mechanisms, particularly during acute proteotoxic stress.

Finally, these mechanisms of lysosomal repair are also reinforced by cytoskeletal remodeling. Following LMP, leucine-rich-repeat kinase 2 (LRRK2) is recruited by RAB29 and phosphorylates RAB8A and RAB10, initiating JIP4-dependent tubulation (61–63, 68). Actin nucleation through the actin-related protein 2/3 complex subunit 2 (ARPC2) and formin-1 maintains curvature and mechanical tension until lipid reconstitution is complete (68). Pathogenic LRRK2 mutations disrupt this choreography and lead to defective repair and dopaminergic neuron vulnerability (61–63).

## Autophagy and lysophagy

**Figure 2:**
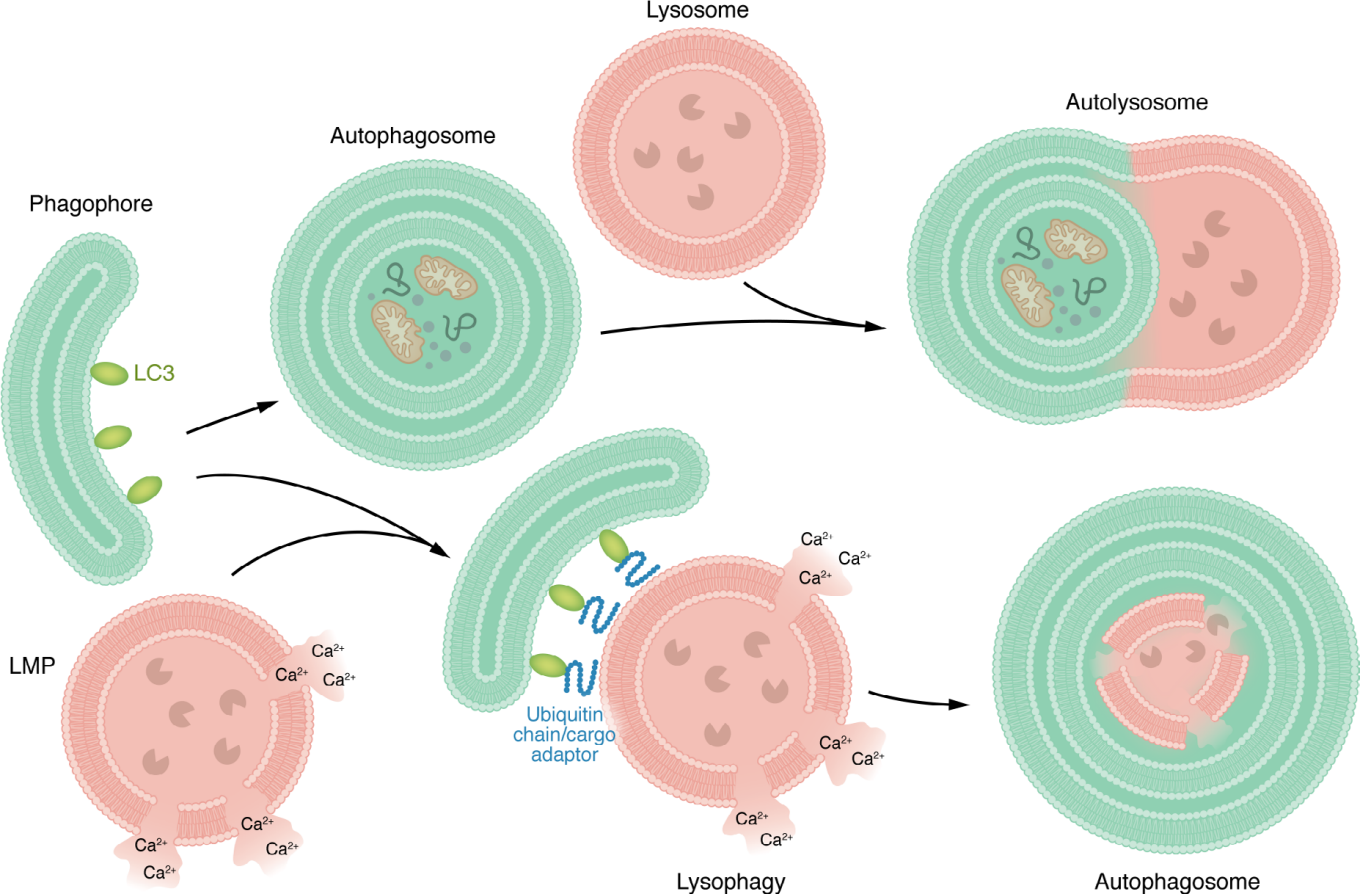
Autophagy and lysophagy pathways. Schematic overview of canonical autophagy and selective lysophagy. Under basal or stress conditions, cytoplasmic material, damaged organelles, and protein aggregates are sequestered by expanding phagophores to form double-membrane autophagosomes. These vesicles fuse sequentially with late endosomes and lysosomes for degradation and recycling. In lysophagy, LMP exposes luminal glycans that recruit galectins, ubiquitin ligases, and autophagy receptors. The phagophore assembly machinery then encapsulates damaged lysosomes for removal. Successful completion restores lysosomal homeostasis, while failure triggers inflammasome activation and neurodegeneration.

To sustain cellular homeostasis and promote survival under stress conditions, eukaryotic cells depend on autophagy, a conserved catabolic pathway that delivers cytoplasmic material to lysosomes for degradation. During this process, portions of cytoplasm, damaged organelles, misfolded proteins, and invading pathogens are enclosed within double-membraned structures termed autophagosomes (69). These form de novo from the ER (70). A defining event in autophagosome formation is the emergence of the phagophore, a crescent-shaped isolation membrane whose molecular assembly remains only partially understood. As the phagophore expands (71), it engulfs cytoplasmic material marked for turnover and eventually seals to generate a mature autophagosome (69). The completed autophagosome then fuses sequentially with late endosomes and lysosomes, where its contents are degraded and recycled to sustain cellular metabolism. This process is choreographed by a highly conserved set of autophagy-related (ATG) proteins that coordinate each stage of autophagosome initiation, maturation, and fusion with the lysosomal compartment (69, 72) (Table 2 and Figure 2).

In selective autophagy, specific cellular components are identified and modified by E3 ubiquitin ligases, which conjugate ubiquitin, a small regulatory protein that signals for degradation, to selected substrates (73, 74). These ubiquitinated cargos are recognized by autophagy adaptor proteins that link them to the autophagic machinery (75). In lysophagy, the selective removal of damaged lysosomes, membrane rupture triggers the accumulation of galectins and ubiquitin on the lysosomal surface (25, 76, 77) (Figure 2). Upon LMP, galectins bind the exposed β-galactosides (33, 34). In parallel, ubiquitination provides a complementary signal for the recognition of damaged lysosomes (73, 78). Following rupture, lysosome-associated membrane protein 1 (LAMP1), and 2 (LAMP2), and transmembrane protein 192 (TMEM192) are modified by ubiquitin (75, 77, 79). These modifications are catalyzed by distinct enzyme complexes, including the SCF^FBXO27^ E3 ligase complex, composed of S-phase–kinase–associated protein 1 (SKP1), cullin-1 (CUL1), and F-box protein 27 (FBXO27) (80), and the cullin-4A–DNA damage–binding protein 1–WD repeat and FYVE domain–containing 1 (CUL4A-DDB1-WDFY1) complex (81). The ubiquitin-conjugating enzyme E2 Q-like 1 (UBE2QL1) also collaborates with lysophagy effectors to promote LAMP1 ubiquitination (78). Once the ubiquitinated and galectin-decorated lysosomal membrane is identified, the phagophore assembly machinery is rapidly mobilized to initiate encapsulation. Galectin-3 and TRIM16 form a signaling platform that recruits the ULK1-ATG13-FIP200 initiation complex (Table 2), marking the site for autophagosome nucleation (82). Consistent with this organizer role, galectin-enriched microdomains help concentrate ubiquitin signals and LC3-binding adaptors, enabling robust lysophagy even when damage is spatially restricted. Specifically, galectin-8 engages the autophagy receptor NDP52, which bridges damaged lysosomes to the microtubule-associated protein 1 light chain 3–positive (LC3-positive) membranes (83). Ubiquitin-binding adaptors, including sequestosome 1 (SQSTM1, also known as p62), tax1-binding protein 1 (TAX1BP1), optineurin (OPTN), and calcium-binding and coiled-coil domain–containing 2 (CALCOCO2, also known as NDP52L) cluster at the lesion, tethering the expanding phagophore to the lysosomal surface (77, 84–86). These adaptors, through their LC3-interacting regions, recruit the ATG5-ATG12-ATG16L1 conjugation system to drive LC3 lipidation and elongation of the isolation membrane around the damaged organelle (87). As the phagophore closes, chaperones such as heat shock protein family B (small) member 1 (HSPB1) and the segregase valosin-containing protein (VCP, also known as p97) remove ubiquitinated membrane remnants, ensuring efficient engulfment (88). The mature autophagosome then fuses with functional lysosomes for degradation of the damaged compartment. This coordinated sequence restores lysosomal homeostasis (25). Recent work also identifies SPART (also known as SPG20) as a lysosomal quality control factor that promotes lysophagy of damaged lysosomes by coordinating early damage recognition with downstream recruitment of autophagy machinery (76). Yet, how cells distinguish repairable from irreversibly damaged lysosomes and how this decision changes with age remains incompletely understood.

After elimination of irreparable organelles, cells restore lysosomal mass through transcriptional regeneration. Calcium efflux through transient receptor potential mucolipin-1 (TRPML1, also known as MCOLN1) on the lysosome membrane activates calcineurin, which dephosphorylates TFEB to drive nuclear translocation and expression of lysosomal and autophagy genes (89). SMAD-specific E3 ubiquitin protein ligase 1–mediated (SMURF1-mediated) calcineurin activation further amplifies TFEB signaling (90). Nuclear TFEB upregulates synthesis of enzymes, transporters, lipid-metabolizing proteins, and factors controlling lysosomal exocytosis, a Ca²^+^-dependent process in which lysosomes fuse with the plasma membrane to release their contents and supply membrane for repair and clearance (7, 89). Whether prolonged TFEB activation remains protective or becomes maladaptive under chronic stress conditions is still debated. In neurons, this renewal pathway replenishes distal lysosomes critical for synaptic maintenance (91, 92). Failure of this regenerative response results in persistent leakage, chronic activation of the NOD LRR- and pyrin domain–containing 3 (NLRP3) and cyclic GMP-AMP synthase (cGAS)–stimulator of interferon gene (STING) pathways, and progressive neuroinflammation (93). Mechanistically, lysosomal damage can promote cytosolic DNA signaling (via mitochondrial or nuclear DNA leakage) to activate cGAS/STING, while STING itself has been proposed to localize to endolysosomal membranes and directly influence lysosomal recovery programs, including TFEB-dependent transcription.

## Lysosomal dysfunction in neurodegenerative diseases

Lysosomal dysfunction represents a common axis of vulnerability across major neurodegenerative disorders (Table 1), where it both precedes and amplifies the accumulation of toxic proteins (17, 23, 51). While extensive evidence supports a role for lysosomal dysfunction in neurodegeneration, the specific contribution of membrane repair and lysosomal quality control pathways to disease initiation versus progression remains an active area of investigation. Most studies to date have documented broad defects in lysosomal degradation, trafficking, or acidification, whereas direct evidence indicating that failure of specific membrane repair pathways such as ESCRT-, PITT-, or lysophagy-mediated mechanisms is causal of disease is still emerging.

When lysosomal damage becomes chronic or exceeds the capacity for repair and lysophagy, it can activate regulated cell death pathways that directly contribute to neuronal loss (10). Extensive lysosomal membrane permeabilization allows the cytosolic release of cathepsins, redox-active iron, and other hydrolases that trigger mitochondrial dysfunction, caspase activation, lipid peroxidation, and inflammatory signaling. Depending on cellular context, these events can engage apoptosis, necroptosis, pyroptosis, or ferroptosis, positioning lysosomes as central hubs that link metabolic stress to cell fate decisions (20–22). In neurons, which rely heavily on lysosomal integrity for long-term proteostasis, persistent lysosomal leakage lowers the threshold for maladaptive cell death, thereby providing a mechanistic bridge between organelle dysfunction and progressive neurodegeneration.

The inability to maintain lysosomal integrity, acidification, or repair capacity directly correlates with neuronal loss in Alzheimer disease (AD) (43), PD (94), amyotrophic lateral sclerosis/frontotemporal dementia (ALS/FTD) (20), and Huntington disease (HD) (95). These findings suggest that impaired lysosomal repair, replacement, and regeneration pathways may act as important modulators of neuronal resilience rather than sole drivers of neurodegenerative pathology.

### AD.

In AD, lysosomal impairment arises early, often preceding the extracellular accumulation of amyloid-β (Aβ) peptides and the intracellular aggregation of tau, a microtubule-associated protein that forms neurofibrillary tangles. Aβ peptides are generated by sequential cleavage of amyloid precursor protein (APP), a transmembrane protein involved in neuronal growth and synaptic repair, by β- and γ-secretases (96, 97). Mutations in presenilin 1 (*PSEN1*) and 2 (*PSEN2*), core components of the γ-secretase complex, alter APP processing and disrupt assembly of the lysosomal vacuolar ATPase (V-ATPase), impairing endolysosomal acidification, hydrolase maturation, and proteolytic efficiency (98–105).

Aβ and tau pathology also compromise the blood-brain barrier (BBB), increasing its permeability to apolipoprotein B–bound (ApoB-bound) cholesterol, which is typically restricted to the peripheral circulation (106, 107). The influx of peripheral cholesterol into the brain perturbs neuronal endolysosomal structure and function, producing enlarged, deacidified lysosomes with reduced degradative capacity (108, 109).

Furthermore, apolipoprotein E (ApoE), particularly the ε4 isoform, modulates lipid trafficking and lysosomal proteolysis in glial cells, further contributing to impaired clearance and neuroinflammation (110–112).

Another hallmark of AD is the accumulation of cathepsin-positive but incompletely digested autophagic vacuoles within dystrophic neurites, indicating inefficient fusion and maturation of autophagosomes into degradative lysosomes (113, 114).

Tau fibrils internalized by neurons and astrocytes accumulate within lysosomes, causing swelling, deacidification, and recruitment of ESCRT machinery; this nanoscale membrane injury promotes cytosolic tau nucleation at the lysosomal surface, suggesting that lysosomal damage may actively initiate tau aggregation (115).

Persistent mTORC1 activity and impaired TFEB/transcription factor binding to IGHM enhancer 3 (TFE3) signaling in AD further suppress lysosomal biogenesis and clearance (116, 117), while defective axonal trafficking causes lysosome accumulation in dystrophic neurites, amplifying local proteostatic collapse (11).

In addition, APP C-terminal fragments have been reported to directly perturb endolysosomal/lysosomal physiology, suggesting that APP-derived species can drive lysosomal stress upstream of overt plaque pathology (118).

Finally, elevated ceramide species in human AD cortices indicate persistent activation of lipid-based repair pathways, underscoring lysosomal injury as an initiating lesion rather than a downstream consequence (119).

At the mechanistic level, these changes are consistent with reduced ESCRT-mediated membrane repair capacity, impaired TFEB-dependent lysosomal renewal, and chronic lipid-driven membrane stress that may overwhelm may overwhelm membrane repair pathways involving the conversion of sphingomyelin to ceramide (sphingomyelin–ceramide).

### PD.

Lysosomal failure is both genetically and mechanistically central to the pathogenesis of PD (94, 120). Mutations in *GBA1*, which encodes the lysosomal enzyme β-glucocerebrosidase (GCase) responsible for hydrolyzing glucosylceramide into glucose and ceramide, reduce enzymatic activity and lead to the accumulation of glucosylceramide and related glycolipids (121–123). These lipid deposits destabilize lysosomal membranes and impair protease function. The resulting lipid imbalance promotes the aggregation of α-synuclein (encoded by *SNCA*), a presynaptic protein that normally regulates synaptic vesicle trafficking but, when misfolded, accumulates in lysosomes and further disrupts autophagic flux (124–126). Excess α-synuclein interferes with SNARE-mediated autophagosome-lysosome fusion, exacerbating autophagic inefficiency (124), while lysosomal exocytosis of α-synuclein aggregates contributes to extracellular propagation of pathology (127).

Further genetic evidence includes the finding that pathogenic mutations in *LRRK2*, which encodes a large multidomain kinase that phosphorylates Rab GTPases involved in vesicle trafficking, alter endolysosomal transport and lysosomal repair dynamics (61–63). Loss of ATP13A2 (*PARK9*), a lysosomal P-type ATPase that regulates polyamine and metal ion homeostasis, increases oxidative stress and impairs autophagic clearance (128–130). Finally, defective lysosomal acidification caused by mutations in transmembrane protein 175 (*TMEM175*), a lysosomal potassium/proton channel that maintains luminal pH, and vacuolar protein sorting-associated protein 35 (*VPS35*), a core component of the retromer complex that mediates cargo recycling, further compromises hydrolase activity and enzyme trafficking (131–137).

Together, these events create a feed-forward cycle of defective degradation and neuroinflammation driving neuronal vulnerability. These defects are consistent with impaired lipid-dependent membrane repair, including sphingomyelin–ceramide turnover and ATG9A-associated lipid exchange pathways, as well as altered lysophagy efficiency and Rab-regulated repair dynamics.

### ALS and FTD.

In ALS and FTD, diverse genetic mutations converge on lysosomal and autophagic dysfunction as a central pathogenic mechanism (17, 138). Progranulin (encoded by *GRN*), a secreted glycoprotein that is processed into granulin peptides within lysosomes, regulates lysosomal acidification, hydrolase trafficking, and membrane stability. Loss-of-function mutations in *GRN* lead to progranulin deficiency, which reduces lysosomal acidity and cathepsin activity, promoting the accumulation of ubiquitinated protein aggregates in the cytoplasm of neurons and glial cells (139–141). Hexanucleotide repeat expansions in chromosome 9 open reading frame 72 (*C9orf72*), the most common genetic cause of both ALS and FTD, cause two major pathogenic outcomes. First, decreased levels of the C9orf72 protein, which normally forms a complex with SMCR8 and WD repeat domain 41 (WDR41) to control endosomal trafficking and autophagy initiation, impair s lysosomal biogenesis and vesicle maturation (142). Second, repeat-associated non-AUG (RAN) translation of the expanded GGGGCC repeats generates toxic dipeptide repeat proteins (DPRs), including poly(GA), poly(GR), and poly(PR) that accumulate in the cytoplasm and nucleus. These DPRs disrupt lysosomal membranes, inhibit proteasome function, and interfere with nucleocytoplasmic transport by binding nuclear pore components (143–146).

Additional ALS/FTD-linked mutations further highlight the centrality of the autophagy-lysosome pathway. Mutations in VCP, an AAA+ ATPase that extracts ubiquitinated substrates from membranes and facilitates autophagosome maturation, impair degradation of damaged organelles (147). Changes in charged multivesicular body protein 2B (*CHMP2B*), a component of ESCR7-III, interfere with endosomal sorting and lysosomal membrane repair (148–150). Loss-of-function variants in TANK-binding kinase 1 (*TBK1*), a serine/threonine kinase that phosphorylates autophagy receptors OPTN and SQSTM1, weaken selective autophagy and the clearance of damaged mitochondria and lysosomes (151–153). Persistent lysosomal stress in ALS/FTD models induces chronic activation of the transcription factors TFEB and TFE3, which drive lysosomal and autophagy gene expression as a compensatory response to degradation failure, although whether prolonged activation becomes maladaptive remains under investigation (154). Furthermore, increased galectin-3 levels in patients with FTD highlight its prominent role in neuroinflammatory signaling that may influence disease pathogenesis (155).

Together, these events create a feed-forward cycle of defective degradation and neuroinflammation driving neuronal vulnerability. Several ALS/FTD-linked mutations, particularly those affecting ESCRT components and autophagy regulators, suggest a convergence on impaired membrane repair, inefficient lysophagy, and dysregulated TFEB/TFE3-mediated transcriptional adaptation.

### HD.

HD, caused by a CAG trinucleotide repeat expansion in the huntingtin gene (*HTT*), is characterized by progressive motor, cognitive, and psychiatric decline (156, 157). The mutant huntingtin protein (mHTT) contains an expanded polyglutamine (polyQ) tract that confers toxic gain-of-function properties and disrupts multiple facets of the lysosome-autophagosome network (95, 158). Under physiological conditions, wild-type HTT scaffolds autophagy and vesicular transport by interacting with HTT-associated protein 1 (HAP1), RAB7, and dynein, thereby mediating retrograde trafficking of autophagosomes and lysosomes along microtubules (159, 160). The mutant form impairs these interactions, leading to defective autophagosome recognition and inefficient transport of lysosomes to distal neuronal compartments, where degradation demand is highest (161, 162). mHTT also perturbs mTORC1 signaling at the lysosomal membrane, locking mTORC1 in an inactive configuration that suppresses TFEB activation and lysosomal gene transcription (163, 164).

Perturbations in lipid composition further compromise lysosomal membrane integrity and repair. In HD, phospholipid and sphingolipid composition is altered (including defective de novo sphingolipid synthesis) and cholesterol homeostasis is dysregulated, with accompanying changes in membrane fluidity; such lipid shifts are expected to stiffen membranes (e.g., via cholesterol/ceramide enrichment) and impede curvature-dependent lysosomal fusion/repair (165–169).

Moreover, mHTT interferes with the activity of RAB5 and RAB7, small GTPases that regulate endosome-lysosome maturation and fusion, resulting in stalled autophagic flux (159, 170).

Recent HD studies further link lysosomal dysfunction to altered galectin-3 signaling and dysregulation of TFEB/TFE3-controlled lysosomal programs, supporting lysosome-centered stress responses as disease modifiers (95, 171, 172).

These changes are consistent with compromised lysosomal renewal and defective recovery from membrane stress, potentially involving reduced TFEB activation, impaired lysophagy, and lipid composition changes that weaken sphingolipid-dependent repair capacity.

### Lysosomal storage disorders.

Lysosomal storage disorders (LSDs) comprise a group of more than 70 inherited disorders caused by mutations affecting lysosomal enzymes, transporters, or structural components, resulting in the accumulation of undegraded substrates and progressive cellular toxicity (173, 174). Among these, the neuronal ceroid lipofuscinoses (NCLs), caused by mutations in CLN genes such as *CLN1* (also known as *PPT1*), *CLN2* (also known as *TPP1*), *CLN3*, and *CLN6* represent prototypical examples in which impaired lysosomal proteolysis and membrane trafficking lead to progressive neurodegeneration (175–179). Deficiency of GCase (*GBA1*) in Gaucher disease leads to accumulation of glucosylceramide and glucosylsphingosine, perturbing lysosomal membrane composition and trafficking (180–184).

In Niemann-Pick disease types A and B, loss of acid sphingomyelinase due to *SMPD1* mutations causes sphingomyelin storage, whereas Niemann-Pick type C (NPC), resulting from *NPC1* or *NPC2* defects, impairs cholesterol and glycosphingolipid export, leading to unesterified cholesterol accumulation and defective endolysosomal motility (185, 186). Other LSDs, such as Tay-Sachs (*HEXA* mutation) and Fabry disease (*GLA* mutation), illustrate how deficits in ganglioside and glycosphingolipid degradation converge on lysosomal dysfunction and neurotoxicity (187).

Across these conditions, substrate overload distorts endolysosomal trafficking and acid hydrolase maturation, destabilizes LAMP2A, and thereby suppresses chaperone-mediated autophagy (188, 189). This disrupts lysosomal Ca²^+^ signaling through TRPML1, as seen in mucolipidosis type IV (190–192). Transcriptional responses driven by TFEB and TFE3 initially upregulate lysosomal biogenesis, but sustained activation may in certain contexts contribute to metabolic exhaustion and persistent inflammation, and its long-term impact remains under active investigation (193). Furthermore, recent evidence shows that STING acts as a lysosomal proton channel that directly activates TFEB under lysosomal stress, linking innate immune sensing to the transcriptional recovery of lysosomal function in storage disorders (194).

Thus, LSDs illustrate how impaired lipid homeostasis, defective repair, and failed autophagic adaptation produce a lysosomal fragility that mechanistically parallels the dysfunction seen in more common neurodegenerative diseases.

## Organelle crosstalk, system integration, and future perspectives

**Figure 3:**
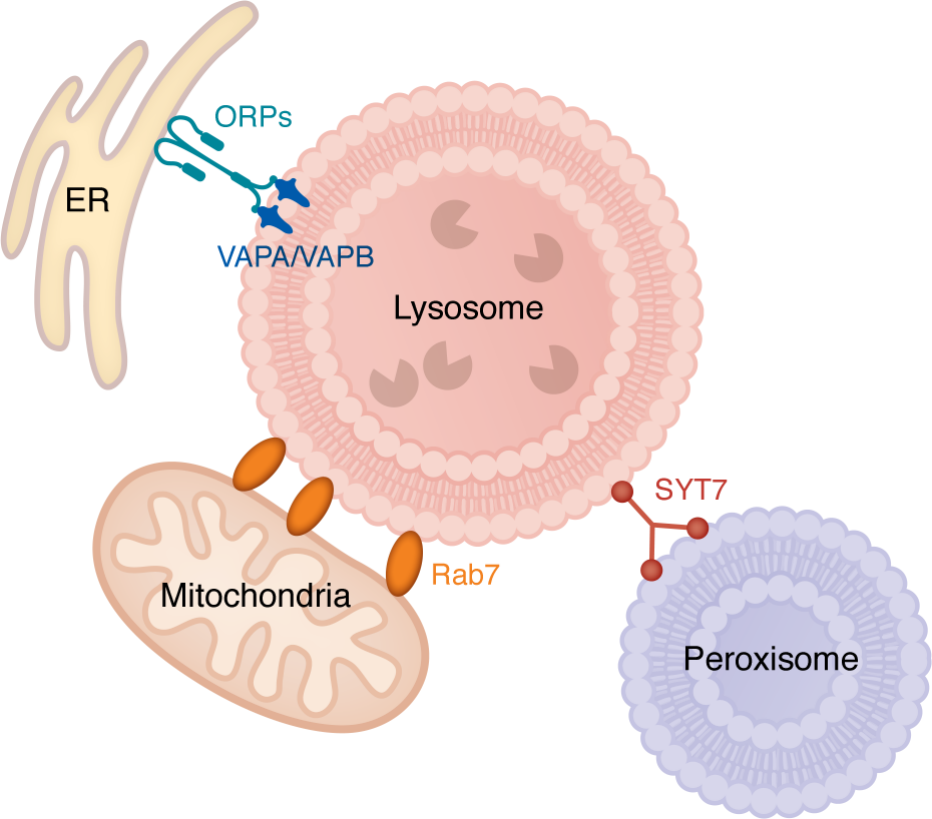
Lysosomal inter-organelle contact sites. Lysosomes establish membrane contact sites with multiple organelles to coordinate metabolism and stress responses. At ER-lysosome interfaces, VAPA and VAPB engage ORPs to mediate cholesterol and phosphatidylserine exchange essential for membrane maintenance and repair. Mitochondria-lysosome contacts organized by Rab7 regulate calcium and lipid transfer, linking degradative capacity with mitochondrial energy supply. Peroxisome-lysosome junctions involving synaptotagmin 7 (SYT7) enable bidirectional lipid trafficking critical for plasmalogen and cholesterol homeostasis. Disruption of these connections impairs lipid balance and lysosomal positioning, sensitizing neurons to metabolic and oxidative stress.

Lysosomes exist within an integrated network of organelles that collectively sustain neuronal metabolism and stress resilience. Physical membrane contact sites connect lysosomes with other intracellular organelles, including the ER, mitochondria, peroxisomes, and the plasma membrane, creating a multidirectional circuit for lipid exchange, calcium signaling, and spatial positioning (195–198) (Figure 3). These dynamic contacts enable neurons to adapt rapidly to metabolic and mechanical stress, ensuring coordination with cellular energetic status. However, the molecular regulation of these contact sites and how their dynamics are altered during aging or disease remain incompletely characterized.

At ER-lysosome interfaces, ER-resident VAPA and VAPB engage members of the ORP family, such as ORP9, ORP10, and ORP11, to mediate bidirectional transfer of cholesterol and PS in exchange for PI4P (48, 49) (Figure 3). This lipid flow maintains lysosomal membrane composition and provides precursors for the PI4K2A/ATG9A/ARFIP2 repair axis after injury (46, 49). During acute stress, ER tubules dynamically wrap around damaged lysosomes, establishing temporary bridges that deliver lipids and facilitate cholesterol-dependent resealing. Disruption of these tethers through the loss of VAPs or PI4K2A sensitizes cells to LMP, emphasizing the interdependence between lipid metabolism and membrane repair (48, 49).

Mitochondria-lysosome coupling is equally essential for maintaining cellular homeostasis (4, 196). Lysosomes remove dysfunctional mitochondria through mitophagy, while mitochondria supply ATP required for lysosomal acidification (199, 200). These organelles communicate through Rab7- and TBC1D15/17-dependent contact sites that coordinate calcium and lipid exchange (201) (Figure 3). Mitochondrial ROS can oxidize lysosomal membranes, whereas lysosomal rupture releases iron and cathepsins that promote mitochondrial permeabilization and apoptosis (202, 203). This bidirectional toxicity establishes a feed-forward loop that accelerates neuronal degeneration, particularly in PD and ALS (4).

Beyond these bilateral contacts, lysosomes also form tethers with peroxisomes, mediated by ORP1L and synaptotagmin VII (SYT7), enabling cholesterol and phospholipid exchange critical for maintaining plasmalogen and membrane lipid homeostasis (197) (Figure 3). Perturbation of this pathway contributes to the lipid dysregulation observed in neurodegenerative conditions. In addition, lysosome–plasma membrane contact sites regulate calcium-dependent exocytosis required for plasma membrane repair and clearance of aggregated proteins. This process involves SYT7, annexins, and SNARE family members such as vesicle-associated membrane protein 7 (VAMP7) and syntaxin 4 (198). Dysregulated lysosomal exocytosis contributes to the extracellular propagation of α-synuclein and tau aggregates in PD and AD (128).

Together, these diverse lysosomal contact sites, spanning the ER, mitochondria, peroxisomes, plasma membrane, and nucleus, form a spatially organized signaling network that integrates metabolism, membrane repair, and stress responses. Their disruption contributes to the defective lipid homeostasis, impaired clearance, and neuronal vulnerability that characterize multiple neurodegenerative diseases.

## Aging and network decline

Aging leads to a gradual erosion of lysosomal quality control across multiple levels. The precise mechanisms driving this decline and whether they reflect intrinsic lysosomal aging or systemic metabolic changes remain open questions. The lipid composition of the lysosomal membrane shifts toward higher cholesterol and ceramide content, increasing rigidity and susceptibility to rupture (204, 205). Autophagic flux declines as the efficiency of autophagosome-lysosome fusion decreases, while TFEB activation becomes blunted due to persistent mTORC1 signaling and reduced calcineurin activity (206). These cumulative defects result in the buildup of lipofuscin, an autofluorescent aggregate composed of oxidized proteins and lipids that physically obstructs lysosomal degradation (207).

Mitochondrial output also wanes with age, reducing ATP availability for proton pumps and compromising lysosomal acidification (208). The number and stability of ER-lysosome and mitochondria-lysosome contact sites decline, limiting lipid and calcium transfer and weakening recovery from transient damage (209). This progressive uncoupling of organellar networks may underlie the early decline in neuronal proteostasis observed during aging, preceding overt neurodegeneration.

## Therapeutic perspectives

Growing mechanistic insight into lysosomal homeostasis is reshaping therapeutic strategies for neurodegenerative diseases. Instead of focusing solely on downstream protein aggregates, emerging approaches aim to restore the lysosome’s intrinsic capacity to repair, replace, or regenerate itself (26, 210). However, although targeting lysosomal repair and regeneration pathways is conceptually promising, further work is needed to determine whether modulation of these mechanisms can alter disease trajectories in human patients.

Enhancing repair capacity represents one avenue. Small molecules that stabilize lysosomal membranes, modulate cholesterol distribution, or promote sphingomyelin–ceramide turnover have shown protective effects in models of neurodegeneration (211–213). Compounds that enhance ESCRT recruitment or boost lipid exchange through the PITT/ATG9A pathway may strengthen intrinsic repair mechanisms.

Reinforcing lysosomal clearance is another strategy. Pharmacological activation of TFEB or TFE3, via mTORC1 inhibition or calcineurin stimulation, upregulates genes that govern lysosomal biogenesis, autophagy, and lipid metabolism (214, 215). In experimental models of AD and PD, TFEB activation accelerates aggregate removal and improves neuronal viability (216, 217).

Targeting LRRK2 signaling holds promise for familial and sporadic PD. Selective LRRK2 kinase inhibitors normalize Rab phosphorylation, restore lysosomal trafficking, and are currently undergoing clinical testing (218, 219).

For LSDs and GBA1-associated PD, enzyme replacement and small-molecule chaperone therapies, such as ambroxol, improve substrate clearance and restore membrane stability (220, 221).

Finally, modulating lysosomal exocytosis may provide an adaptive outlet for cells under chronic stress. Controlled stimulation can aid in the removal of undegraded material and plasma membrane repair, although excessive activation risks propagating toxic aggregates and inflammatory signals.

Developing biomarkers of lysosomal health will be essential to monitor therapeutic efficacy and guide precision interventions.

## Conclusion

Lysosomal homeostasis defines neuronal longevity by integrating stress sensing, repair, and regeneration into a unified survival program. While current evidence does not establish lysosomal repair failure as a universal initiating event in neurodegeneration, growing data support its role as a critical determinant of neuronal vulnerability and disease progression. Across neurodegenerative diseases, the failure of these adaptive mechanisms precipitates proteostatic collapse and inflammation. The convergence of ESCRT-mediated mechanics, lipid-based membrane repair, and TFEB-dependent transcriptional renewal reveals a common molecular architecture that underpins neuronal resilience. Restoring this triad through targeted modulation of lipid metabolism, membrane repair capacity, and transcriptional control offers a coherent strategy to halt neurodegeneration at its origin. Future work aimed at defining damage thresholds, pathway coordination, and neuron-specific vulnerabilities will be essential for translating these insights into therapeutic strategies.

## Funding support

- Cancer Research UK grant CC2134 (to the Francis Crick Institute).
- UK Medical Research Council grant CC2034 (to the Francis Crick Institute).
- Wellcome Trust grant CC2134 (to the Francis Crick Institute).

## Figures and Tables

**Table 2:**
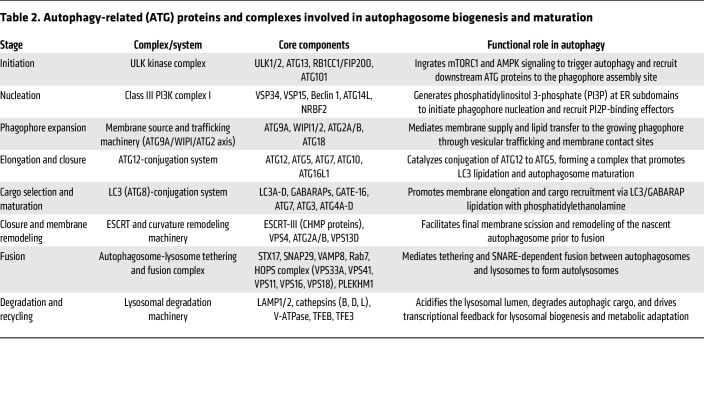
Autophagy-related (ATG) proteins and complexes involved in autophagosome biogenesis and maturation

**Table 1:**
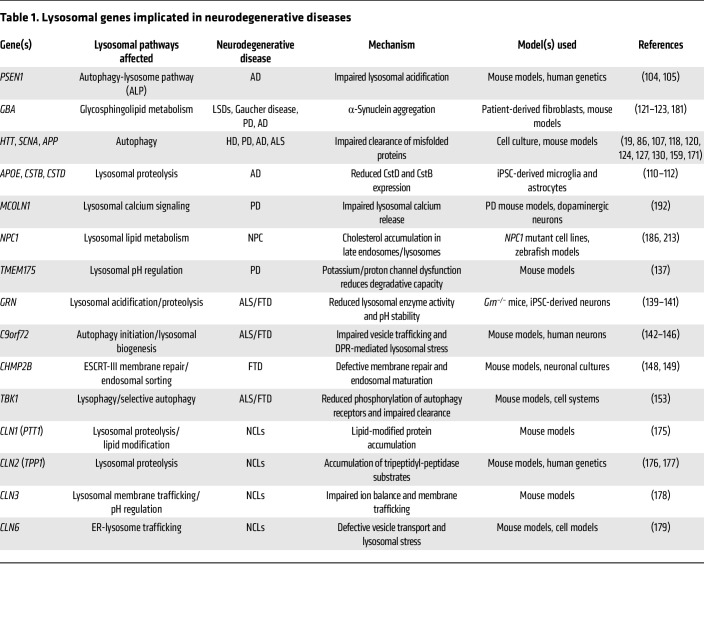
Lysosomal genes implicated in neurodegenerative diseases

## Version 1 04/01/2026

Electronic publication
